# Myb proteins inhibit fibroblast transformation by v-Rel

**DOI:** 10.1186/1476-4598-5-54

**Published:** 2006-11-02

**Authors:** Shu-ling Fu, Brigitte Ganter, Joseph S Lipsick

**Affiliations:** 1 Departments of Pathology and Genetics, Stanford University School of Medicine, 300 Pasteur Drive, Stanford, CA 94305-5324, USA

## Abstract

Genes that cause cancer have been divided into two general classes – oncogenes that act in a dominant fashion to transform normal cells into a malignant state, and tumor suppressor genes that act in a dominant fashion to prevent such transformation. In this report, we demonstrate that both the v-*myb *retroviral oncogene, which causes leukemic transformation of hematopoietic cells, and the c-*myb *proto-oncogene can also function as inhibitors of fibroblast transformation by the v-*rel *oncogene. These results imply that the *myb *genes can function either as oncogenes or as tumor suppressors in different cellular contexts.

## Background

The oncogenic transformation of normal cells of vertebrates is a multi-step process in which mutations accumulate in two classes of cellular genes, oncogenes and tumor suppressor genes [1]. Oncogenes are altered forms of normal cellular proto-oncogenes that act in a dominant fashion to convert normal cells into a malignant state. In contrast, tumor suppressor mutants act in a recessive fashion within the cell and, in general, one wild type copy of a tumor suppressor gene is sufficient to inhibit transformation.

The v-*myb *oncogene of the avian myeloblastosis virus is unusual because unlike other known oncogenes, it causes only leukemias in animals and transforms only hematopoietic cells and not fibroblasts in culture [2]. Members of the Myb protein family bind to specific DNA sequences, can directly regulate gene expression, and have been highly conserved during eukaryotic evolution [3]. The *myb *oncogene has previously been shown to cooperate with the v-*ets *oncogene in the transformation of hematopoietic cells [4]. Indeed, the *ets *gene family was initially discovered because of the presence of both v-myb and v-ets within a single acutely transforming retrovirus, the E26 leukemia virus [5].

The v-*rel *oncogene of the avian reticuloendotheliosis virus strain T (REV-T) causes a malignant proliferation of immature lymphoid cells in animals and can transform both lymphoid and fibroblastic cells in culture [6]. However, fibroblast transformation by this virus is somewhat weaker than that caused by a variety of other oncogenes [7]. Members of the Rel protein family include *Drosophila *Dorsal and vertebrate NF-kB, and like Myb, these proteins bind to specific DNA sequences and can directly regulate gene expression [8]. In order to test whether v-*myb *and c-*myb *could cooperate with v-*rel *in oncogenic transformation of hematopoietic cells, we constructed a series of avian retroviruses that coexpress either one or both of these oncogenes. Quite unexpectedly, we found that v-*myb *and c-*myb *suppress fibroblast transformation by v-*rel*.

## Results and discussion

### v-Myb and c-Myb inhibit fibroblast transformation by v-Rel-ER

**Figure 1:**
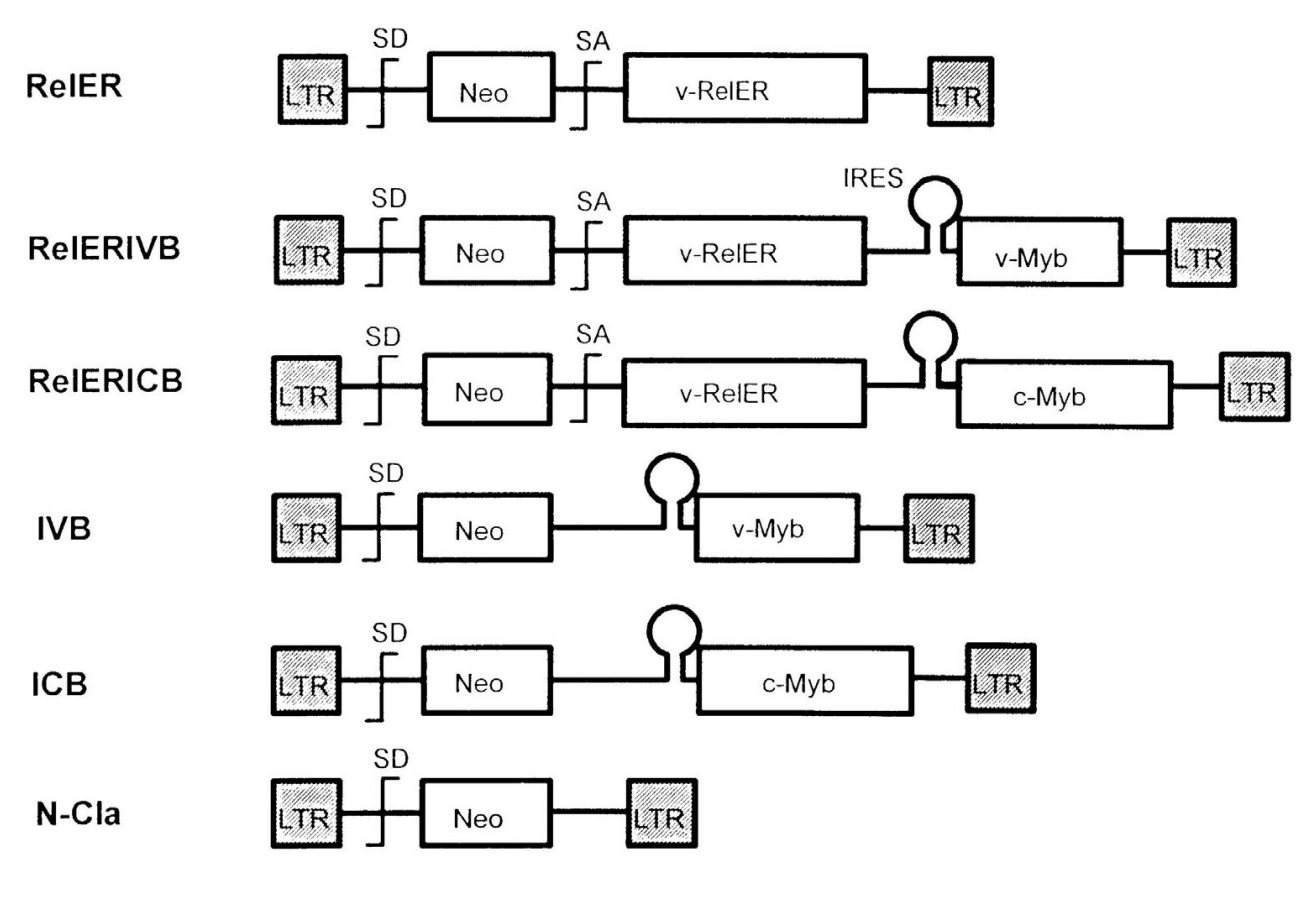
Structure of viruses used in this study. The long terminal repeats (LTR) were derived from MAV-1. Also shown are the splice donor sites (SD), splice acceptor sites (SA) and the internal ribosomal entry sites (IRES) used to express two or three genes from a single virus.

To insure the efficient production of both Rel and Myb proteins in the same cell by viruses containing two oncogenes, we have used an internal ribosomal entry site (IRES) from the encephalomyocarditis virus to permit translation of both proteins from a single mRNA (Figure 1) [9]. This strategy of coexpression has previously been shown to be more efficient than the use of retroviruses that depend on internal promoters or alternative splicing to produce two different gene products [10]. All of these viruses were derived from the myeloblastosis associated virus type 1 (MAV-1), the natural helper virus for AMV, and also included the dominantly selectable *neo *gene [11,12]. In addition, we used an estrogen-inducible form of the v-Rel protein (v-Rel-ER) so that we could examine whether Rel activity was required for the maintenance of transformation in the presence or absence of Myb proteins [13].

**Figure 2:**
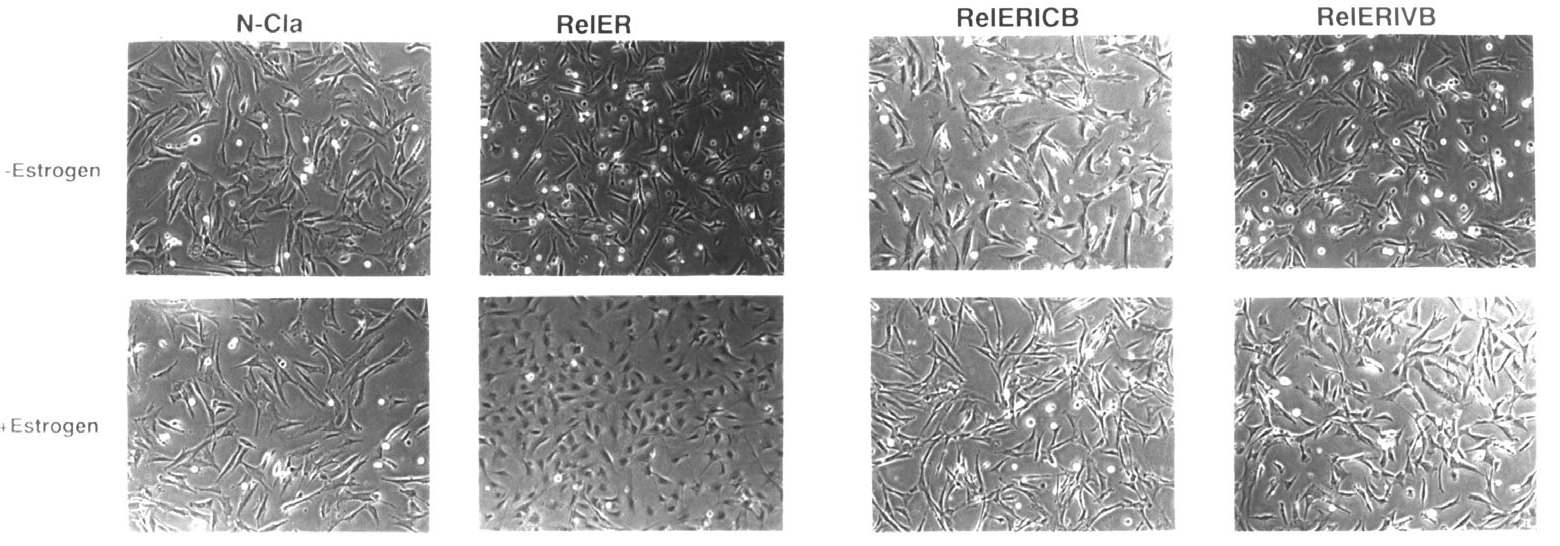
Myb proteins suppress transformation by v-*rel*. Primary cultures of chicken embryo fibroblasts were co-transfected with the indicated proviruses and the MAV-1 helper virus. Two days later G418 (200 ug/ml) was applied to select for the expression of the *neo *gene. The morphology of the resulting G418-resistant cells was observed by phase contrast microscopy. The cells infected with the Rel-ER virus have a distinct transformed morphology in the presence but not in the absence of estrogen (10^-6 ^M). This transformed morphology was suppressed by c-*myb *(Rel-ER-ICB) or v-*myb *(Rel-ER-IVB).

Plasmid DNAs containing the replication defective proviruses shown in Figure 1 were each cotransfected along with the MAV-1 helper virus into primary cultures of chicken embryonic fibroblasts. Two days later, cells were treated with G418 to select for the expression of the defective proviruses, each of which contained the *neo *gene. As shown previously, the v-Rel-ER protein was capable of transforming primary cultures of chicken embryo fibroblasts in an estrogen-dependent fashion (Figure 2). The control vector containing only the *neo *gene (N-Cla) had no effect on fibroblast growth or morphology. As previously reported, v-Myb alone was incapable of transforming chicken embryonic fibroblasts (not shown).

Somewhat surprisingly, the virus that encoded both the v-Rel-ER and v-Myb proteins was incapable of causing fibroblast transformation either in the presence or absence of estrogen (Figure 2). The v-Myb protein is a doubly truncated form of the normal c-Myb protein that has also sustained a number of amino acid substitutions relative to c-Myb [14]. In order to determine whether the ability of v-Myb to suppress fibroblast transformation by v-Rel is a result of these alterations in the v-Myb protein, we constructed similar viruses that expressed either c-Myb alone, or both v-Rel-ER and c-Myb (Figure 1). As was observed with v-Myb, c-Myb itself was incapable of transforming chicken embryonic fibroblasts (not shown). Furthermore, c-Myb was able to completely suppress transformation by v-Rel-ER in a fashion similar to v-Myb (Figure 2). These results demonstrate that the v-*myb *gene, which is capable of oncogenically transforming macrophage precursors and causing monoblastic leukemias in vivo, can act in an opposing fashion by suppressing fibroblast transformation by the v-*rel *oncogene. Furthermore, the c-*myb *gene which can also cause the outgrowth of myelomonocytic cells in culture [15,16], behaves similar to v-*myb *in suppressing transformation by v-*rel*.

### Myb proteins inhibit the actin cable reorganization induced by v-Rel-ER

**Figure 3:**
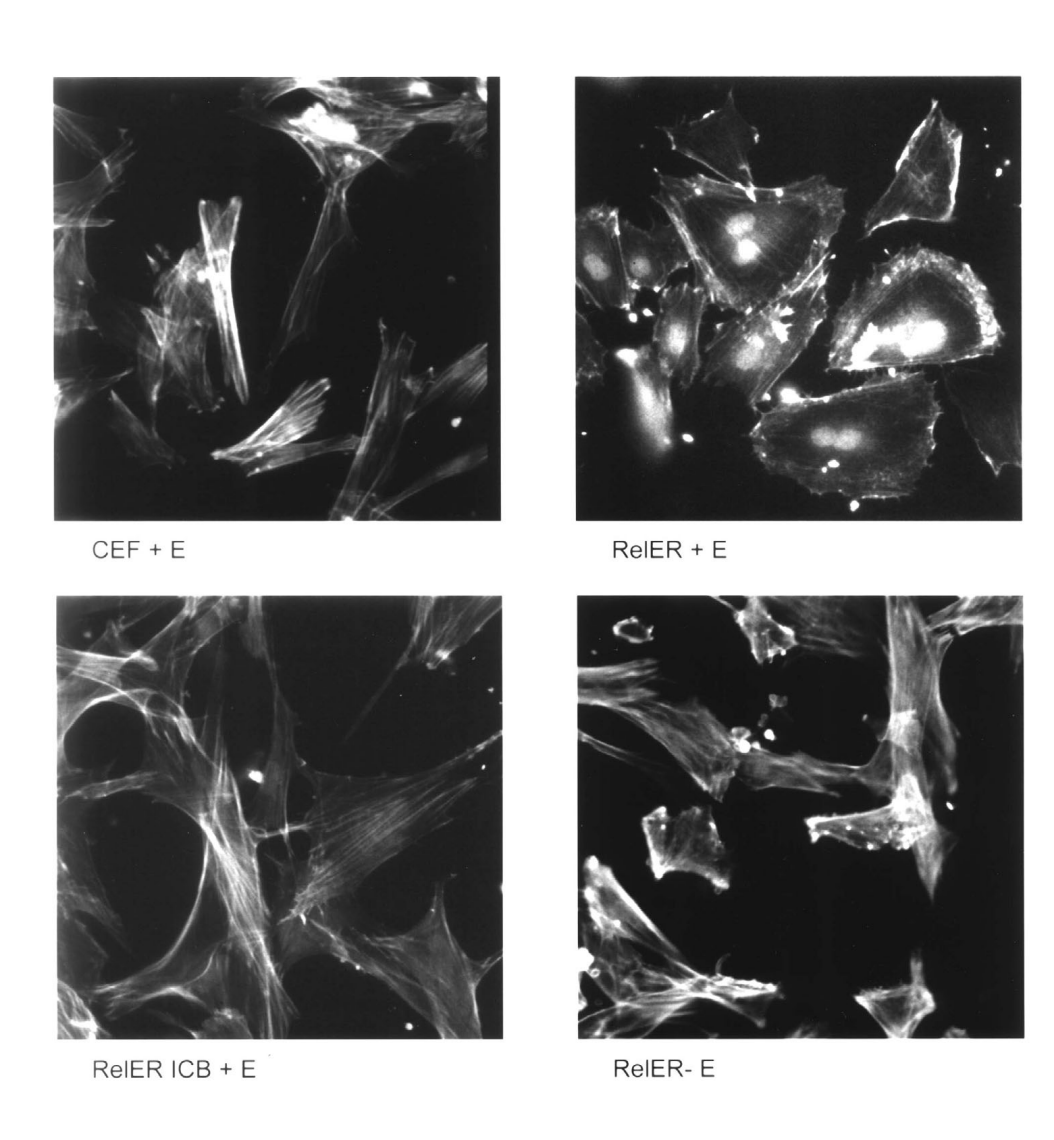
Myb proteins suppress cytoskeletal reorganization by v-*rel*. Primary chicken embryo fibroblasts (CEF) and cells infected with the indicated viruses were stained with fluorescent phalloidin to decorate intracellular actin filaments. The cells infected with the Rel-ER virus showed a loss of actin cables and increased cortical actin in the presence of estrogen (+E2). Estrogen alone had no detectable effect on uninfected cells. Co-expression of c-*myb *(Rel-ERICB) suppressed this cytoskeletal reorganization.

In addition to the altered cellular morphology induced by v-Rel-ER in the presence of estrogen, histochemical staining with fluorescently labeled phalloidin revealed a dramatic reorganization of cytoskeletal actin in the presence but not in the absence of estrogen (Figure 3). The actin stress fibers seen in control fibroblasts were replaced by a dense accumulation of cortical actin adjacent to the plasma membrane in the v-Rel-ER cells in the presence of estrogen. This change is a hallmark of fibroblast transformation by the v-rel oncogene [17]. Both the transformed cellular morphology and the actin redistribution could be reversed by the withdrawal of estrogen from the culture medium despite the continued presence of the v-Rel-ER protein, as previously shown [13]. Consistent with the inhibition of Rel-induced transformation by c-Myb, no reorganization of the actin cytoskeleton was observed in cells infected with the Rel-ER/c-Myb virus even when estrogen was added to the culture medium (Figure 3). Similarly, no reorganization of the actin cytoskeleton was observed in cells infected with the Rel-ER/v-Myb virus (data not shown).

### Protein expression by the dicistronic Rel/Myb viruses

**Figure 4:**
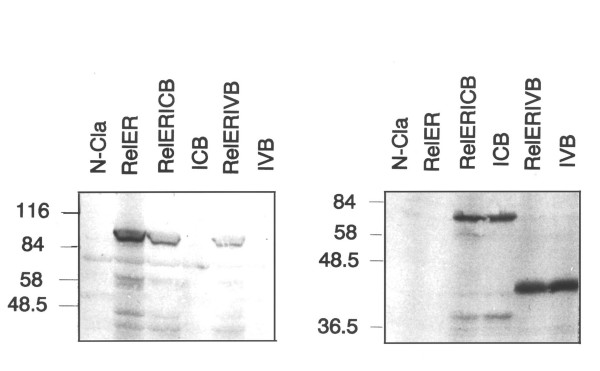
Myb proteins do not prevent expression of Rel-ER. Primary chicken embryo fibroblasts infected with the indicated viruses were lysed and analyzed by SDS-PAGE and immunoblotting using either anti-Rel (left panel) or anti-Myb (right panel) antibodies.

One possible explanation for the failure of the two-oncogene viruses to transform chicken embryonic fibroblasts was that the structure of the IRES viruses somehow prevented translation of the v-Rel-ER fusion protein. To address this question, cells infected with each of the viruses shown in Figure 1 were selected with G418 and total protein lysates of these cells were resolved by denaturing polyacrylamide gel electrophoresis. Duplicate gels were blotted onto nitrocellulose membranes and antibodies specific for either the Rel or Myb proteins were used as probes (Figure 4). A protein of the molecular weight predicted for the v-Rel-ER fusion protein was detected in cells infected with all three viruses encoding this protein. In contrast, no Rel proteins were detected by immunoblotting in cells infected with a control virus encoding only the *neo *gene product or in cells infected with viruses encoding only the v-Myb or c-Myb proteins (Figure 4). The v-Myb and c-Myb proteins were clearly detected in cells infected with all four viruses predicted to encode these proteins, but not in cells infected with the viruses encoding only *neo *or v-Rel-ER without v-Myb or c-Myb. These results demonstrate that the v-Rel-ER protein was produced in the presence or absence of v-Myb and c-Myb. Therefore, the inhibition of v-Rel-ER transformation by v-Myb and c-Myb does not appear to be due to a failure of v-Rel-ER protein production.

**Figure 5:**
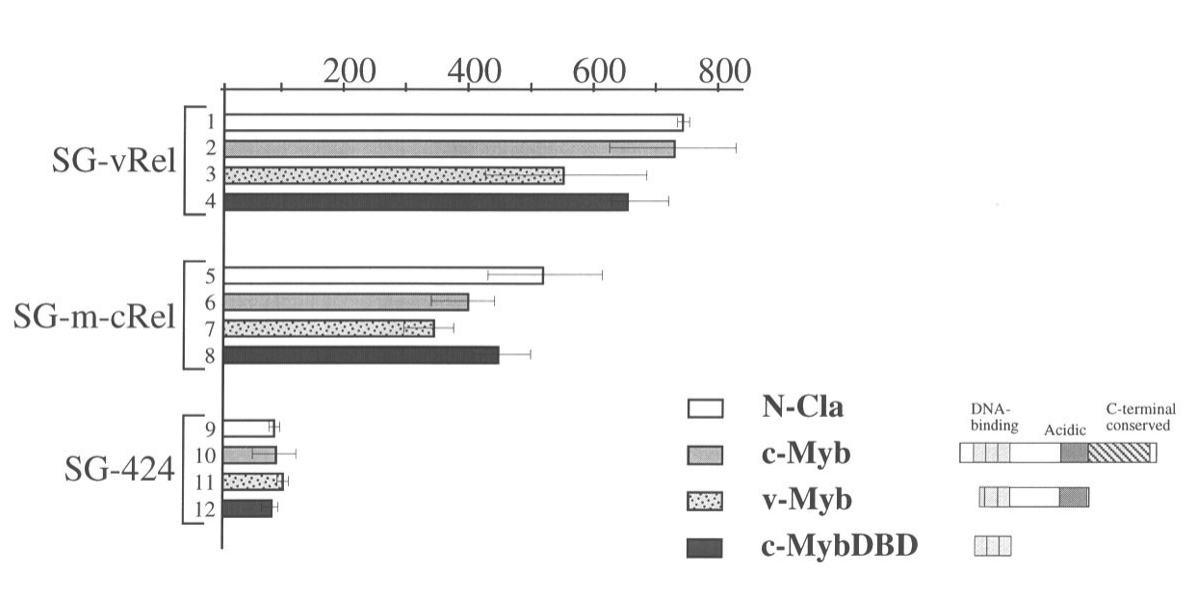
Myb proteins do not inhibit the Rel transcriptional activation domain. QT6 quail fibroblasts were transfected with a GAL4-repsonsive reporter gene, a plasmid producing the indicated GAL4-Rel fusion (SG-424 is a GAL DNA-binding domain only control), and a plasmid producing the indicated Myb protein (N-Cla is a control expression vector; DBD is a DNA-binding domain only control). Transcriptional activation was measured as previously described [18]. Error bars indicated standard errors of the mean.

To investigate the mechanism by which v-*myb *and c-*myb *inhibit fibroblast transformation by v-*rel*, we first performed a series of transient transfections in the quail QT6 fibroblastic cell line with reporter genes containing either Myb or Rel binding sites. Transcriptional activation by v-Myb and c-Myb was unaffected by the presence of v-Rel-ER (data not shown). We were unable to assay transcriptional activation by v-Rel-ER in avian fibroblasts because, as previously reported by others, Rel-responsive reporter genes are highly activated by endogenous Rel-NFkB family proteins in the absence of any exogenous Rel [18]. However, the transcription activity of these endogenous Rel family proteins was unaffected by the presence or absence of Myb proteins (data not shown). Therefore, we examined the effects of v-Myb and c-Myb on transcriptional activation by GAL4-Rel fusion proteins [19]. Both a GAL4-v-Rel and a GAL4-c-Rel fusion protein were able to activate transcription from a reporter gene containing GAL4p binding sites. However, this activation was unaffected by either v-Myb or c-Myb (Figure 5). These results suggest that the Myb proteins do not inhibit transformation by v-Rel by a transcriptional "sequelching" in which an excess of one activation domain limits the availability of a critical coactivator molecule [20]. However, at present we cannot rule out the possibility that Myb proteins specifically interfere with transcriptional regulation by Rel proteins in a manner that cannot be adequately modeled using GAL4-Rel fusion proteins.

### Rel and Myb proteins are associated with one another in nuclear extracts

**Figure 6:**
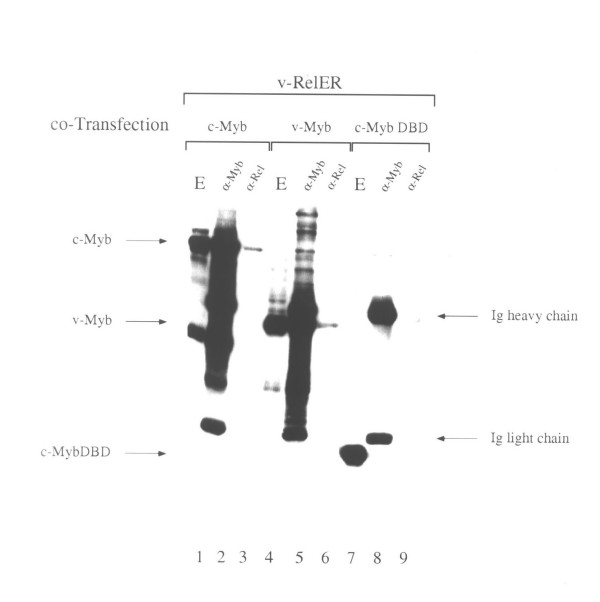
Co-precipitation of Myb and Rel proteins. Quail fibroblasts co-transfected with expression vectors for Rel-ER and the indicated Myb proteins were immunoprecipitated with either anti-Myb or anti-Rel antibodies, then analyzed by SDS-PAGE and immunoblotting using an anti-Myb antibody. A sample of the total extract (E) prior to immunoprecipitation was co-electrophoresed as a control. c-Myb and v-Myb, but not the c-Myb DNA-binding domain alone were co-precipitated with anti-Rel antibodies.

To further investigate the mechanism by which Myb proteins inhibit fibroblast transformation by v-*rel*, we asked whether these proteins associate with one another in cells. For this purpose, QT6 fibroblasts were transfected with expression vectors for v-Rel-ER and c-Myb, v-Myb, or an N-terminal fragment of c-Myb containing only the DNA-binding domain. Lysates of these cells were precipitated with either anti-Myb or anti-Rel antibodies. The precipitated proteins were resolved by denatured gel electrophoresis, transferred to nitrocellulose filters, and then probed with an anti-Myb antibody (Figure 6). As expected, all three Myb proteins could be detected following immunoprecipitation with anti-Myb antibodies (lanes 2, 5, and 8). In addition, both c-Myb and v-Myb were coprecipitated with anti-Rel antibodies in the presence of v-Rel-ER (lanes 3 and 6), whereas the c-Myb DNA-binding domain was not similarly coprecipitated (lane 9). This coprecipitation was not due to cross-reactivity of Myb proteins with anti-Rel antibodies nor did it require the estrogen receptor hormone binding domain, because similar coprecipitation was observed in the presence but not the absence of v-Rel (data not shown). These results demonstrate that the Rel and Myb proteins are capable of forming a complex in cellular extracts. This complex is unlikely to be due simply to the bridging of Rel and Myb proteins by DNA, because the c-Myb DNA binding domain alone did not coprecipitate with Rel proteins.

### Myb genes as inhibitors of oncogenic transformation

Our results demonstrate that v-*myb *and c-*myb*, which can both cause the transformation of hemayopoietic cells, can also function as suppressors of fibroblast transformation by the v-*rel *oncogene. These results suggest that the cell type in which v-*myb *or c-*myb *is expressed appears to determine whether they function as oncogenes or tumor suppressor genes. Because neither v-Myb nor c-Myb are normally present in fibroblasts, one hypothesis is that v-Myb and c-Myb may act as tumor suppressors by dominantly inhibiting the function of B-Myb, a closely related protein which appears to be ubiquitously expressed in all vertebrate cells including in fibroblasts [21].

However, v-Myb and c-Myb do not appear to function as general suppressors of fibroblast transformation. Rather, the mechanism of transformation also plays a role in determining whether Myb proteins function as tumor suppressors, because similar experiments have shown that fibroblast transformation by the v-*myc *oncogene of the MC29 virus is not suppressed by the v-Myb or c-Myb proteins. Furthermore, the *myb *and *rel *oncogenes are not mutually antagonistic in all cells types. In particular, when hematopoietic yolk sac or bone marrow hematopoietic cells are infected with viruses containing both v-*myb *and v-*rel-ER*, transformed myeloid cells grow out that are indistinguishable from cells transformed by v-*myb *alone (data not shown).

A model that could explain these data is that v-Rel and the Myb proteins oppose each other in regulating a common set of genes that are essential for transformation by v-*rel*, whereas the genes essential for transformation by v-*myc *are not affected by Myb proteins. In this regard, gene expression profiling of transformed lymphocytes have suggested that at least some genes may be regulated in common by both Myb and Rel proteins, although many are not [22]. Our data may also explain why v-*myb *is unique among the known retroviral oncogenes in that it does not transform fibroblasts – because in fibroblasts v-*myb *appears to function as an inhibitor of transformation rather than as an oncogene. In addition, our findings may offer some explanation for the paradoxical observation that elevated levels of c-*myb *proto-onoocgene expression are a positive prognostic indicator in human breast cancer [23].

## Conclusion

The v-*myb *oncogene was discovered because of its ability to cause monoblastic leukemia in chickens. Altered forms of the c-myb proto-oncogene cause leukemia and lymphoma in birds and mammals. However, neither v-*myb *nor c-*myb *have been shown to oncogenically transform fibroblasts. We report here that v-*myb *and c-*myb *can inhibit fibroblast transformation by the v-*rel *oncogene, demonstrating that in at least some cellular contexts, v-*myb *and c-*myb *can function as tumor suppressors.

## Methods

### Plasmid constructions

The construction of the N-Cla and N-ICB proviruses has been described previously [16,24]. The N-IVB provirus was constructed by cloning the ClaI-resistant IRES-v-*myb *fragment of SP73-IVB into N-Cla. SP73-IVB itself contains the EcoRI/MscI-resistant IRES fragment of the murine encephalomyocarditis virus (EMCV) and the MscI/XbaI resistant v-*myb *fragment of MT7-MYB in a modified SP73 vector in which the entire polylinker was replaced by a single ClaI site [9,25]. The N-Rel-ER provirus was constructed by cloning the small ClaI-resistant fragment of RCAS-Rel-ER [13] into the ClaI site of a modified N-dGE vector [26] in which the v-*myb *coding sequence but not the splice acceptor site had been removed by digestion with KpnI and ClaI, fill-in with the Klenow fragment of DNA polymerase I and insertion of a ClaI linker. The N-Rel plasmid was constructed in a similar fashion using the small ClaI-resistant fragment of RCAS-REL [17]. The N-Rel-ER-IVB and N-Rel-ER-ICB proviruses were created by ligation of the small ClaI-resistant fragments from N-IVB (v-*myb*) or N-ICB (c-*myb*) into the unique ClaI site of N-Rel-ER which lies downstream of the Rel-ER open reading frame.

### Cell culture and DNA transfections

Primary chicken embryo fibroblasts (CEF) were prepared by trypsinizing the bodies of 7 to 9 day old chicken embryos. These cells were grown in a 37°C, humidified 5% CO_2 _incubator, in Dulbecco's modified essential medium (DMEM) supplemented with glucose (4.5 g/l), 1X MEM nonessential amino acids, 1 mM sodium pyruvate, 2 mM glutamine, streptomycin (100 ug/ml), penicillin (100 U/ml), 2% heat-inactivated chicken serum (56°C, 1 hr), and 8% fetal calf serum. QT6 cells were grown in similar conditions except that 5% fetal calf serum and no chicken serum were added. Where indicated, estradiol was present at a concentration of 1 uM. DNA transfections, luciferase assays, and β-galactosidase assays were performed as described previously [27].

### Actin cable staining

CEFs grown in chamber slides were washed with PBS twice and fixed with lysine-paraformaldehyde-PBS (0.075 M lysine, 0.0375 M sodium phosphate, 2% paraformaldehyde, pH 7.4) for 20 minutes at room temperature. Fixed cells were then washed three times with PBS and stained with rhodamine-phalloidin (Molecular Probes, Inc) for 25 minutes at room temperature. After staining, the cells were washed three times with PBS and overlaid with p-phenylendiamine (PPD)-mounting medium (1%(w/v) PPD, 90 % glycerol, pH 8.5) and visualized by fluorescence microscopy.

### Immunoblotting

Cells were washed once with PBS, scraped off the plates in PBS, centifuged, then lysed in 1X sodium dodecyl sulfate (SDS)-loading buffer and boiled for 5 minutes. Normalized volumes of lysates were subjected to 10% SDS-polyacrylamide gel electrophoresis. Proteins were then transferred to nitrocellulose membranes (BA-S 83, Schleicher & Schuell). Myb expression was detected using a mixture of the Myb 2.2 and 2.7 monoclonal murine antibodies [28]. Rel expression was detected using a polyclonal rabbit antiserum SB66 (1:1000 dilution) kindly provided by Amy Walker and Paula Enrietto. Blots were developed using either goat anti-mouse IgG or goat anti-rabbit IgG conjugated to alkaline phosphotase (Promega), 5-bromo-4-chloro-3 indolylphosphate (BCIP) and nitroblue tetrazolium (NBT) according to the manufacturer's instructions.

### Immunoprecipitation

Quail fibroblasts co-transfected with expression vectors for v-Rel-ER or v-Rel and the indicated Myb proteins were lysed in RIPA buffer without SDS, immunoprecipitated with either mouse the monclonal Myb 2.2 antibody which recognizes a region of v-Myb and c-Myb near the transcriptional activation domain, or the rabbit polyclonal anti-Rel antiserum SB66 using RIPA buffer without SDS and protein G sepharose [29]. Precipitates were then analyzed by SDS-PAGE and immunoblotting using the monoclonal 5E anti-Myb antibody which recognizes the DNA binding domain [30]. A sample of the total extract prior to immunoprecipitation was co-electrophoresed as a control.

## Authors' contributions

SLF and BG conducted all of the experiments described in this study. JSL participated in its design and coordination and helped to draft the manuscript. All authors read and approved the final manuscript.
